# Supplementary material for: Lipophilicity Determination of Antifungal Isoxazolo[3,4-b]pyridin-3(1H)-ones and Their N1-Substituted Derivatives with Chromatographic and Computational Methods
Source: Molecules. 2019 Nov 26;24(23):4311. doi: 10.3390/molecules24234311 (PMC6930598; doi:10.3390/molecules24234311)

Lipophilicity determination of antifungal isoxazolo[3,4-*b*]pyridin-3(1*H*)-ones and their N1-substituted derivatives with chromatographic and computational methods

Krzesimir Ciura[1*], Joanna Fedorowicz[2], Filip Andrić[3], Petar Žuvela[4], Katarzyna Ewa Greber[1], Paweł Baranowski[1], Piotr Kawczak[4], Joanna Nowakowska[1], Tomasz Bączek[4], Jarosław Sączewski[5]**

[1] Department of Physical Chemistry, Faculty of Pharmacy, Medical University of Gdańsk, Al. Gen. J. Hallera 107, 80-416, Gdańsk, Poland
[2] Department of Chemical Technology of Drugs, Faculty of Pharmacy, Medical University of Gdańsk, Al. Gen. J. Hallera 107, 80-416, Gdańsk, Poland
[3] Department for Analytical Chemistry, Faculty of Chemistry, University of Belgrade, Studentski trg 12–16, 11000, Belgrade, Serbia
[4] Department of Pharmaceutical Chemistry, Faculty of Pharmacy, Medical University of Gdansk, Al. Gen. J. Hallera 107, 80-416 Gdańsk, Poland
[5] Department of Organic Chemistry, Faculty of Pharmacy, Medical University of Gdańsk, Al. Gen. J. Hallera 107, 80-416, Gdańsk, Poland

* Correspondence: krzesimir.ciura@gumed.edu.pl
** Current address: Department of Chemistry, National University of Singapore, Singapore 117543, Singapore

Table 1S. Chemical names and structural formulas of the studied of pyrido- and quinolino-isoxazolones

No.	Chemical name	Chemical structure
**1**	isoxazolo[3,4-*b*]quinolin-3(1*H*)-one	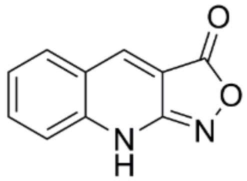
**2**	1-benzylisoxazolo[3,4-*b*]quinolin-3(1*H*)-one	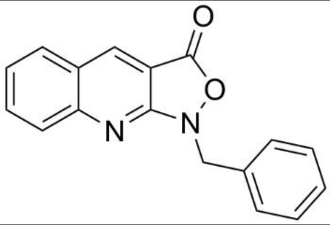
**3**	1-methylisoxazolo[3,4-*b*]quinolin-3(1*H*)-one	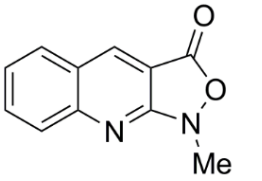
**4**	1-acetylisoxazolo[3,4-*b*]quinolin-3(1*H*)-one	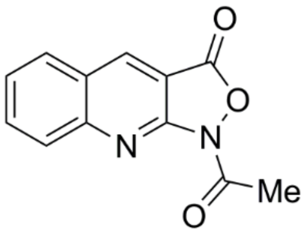
**5**	1-benzoylisoxazolo[3,4-*b*]quinolin-3(1*H*)-one	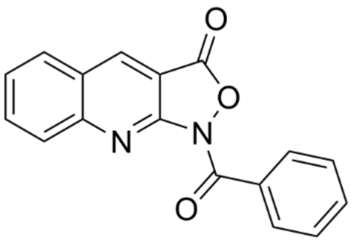
**6**	1-benzyl-4,6-dimethylisoxazolo[3,4-*b*]pyridin-3(1*H*)-one	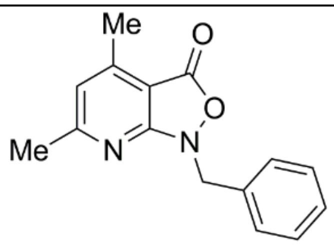

**7**	1-acetyl-4,6-dimethylisoxazolo[3,4-*b*]pyridin-3(1*H*)-one	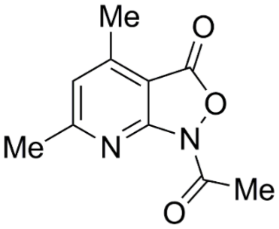
**8**	1-ethyl-4,6-dimethylisoxazolo[3,4-*b*]pyridin-3(1*H*)-one	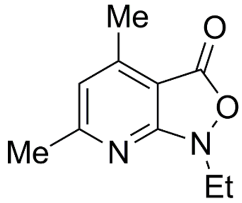
**9**	1-benzoyl-4,6-dimethylisoxazolo[3,4-*b*]pyridin-3(1*H*)-one	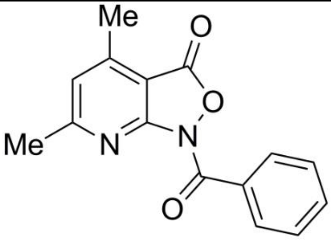
**10**	1,4,6-trimethylisoxazolo[3,4-*b*]pyridin-3(1*H*)-one	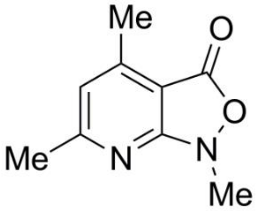
**11**	1-(3,5-dimethoxybenzyl)-4,6-dimethylisoxazolo[3,4-*b*]pyridin-3(1*H*)-one	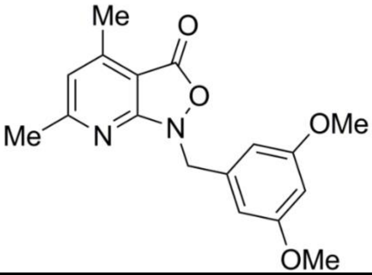
**12**	4,6-dimethyl-1-(methylsulfonyl)isoxazolo[3,4-*b*]pyridin-3(1*H*)-one	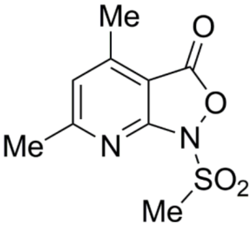
**13**	1-butyl-4,6-dimethylisoxazolo[3,4-*b*]pyridin-3(1*H*)-one	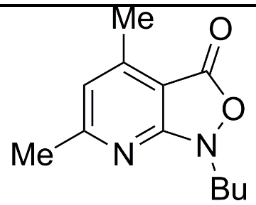
**14**	4,6-dimethylisoxazolo[3,4-*b*]pyridin-3(1*H*)-one	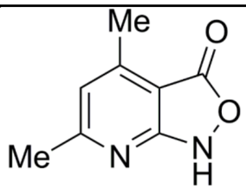

**15**	4,6-dimethyl-1-propylisoxazolo[3,4-*b*]pyridin-3(1*H*)-one	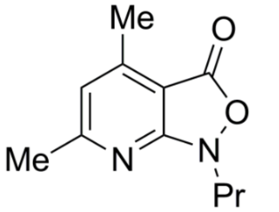
**16**	4,6-dimethyl-1-(prop-2-yn-1-yl)isoxazolo[3,4-*b*]pyridin-3(1*H*)-one	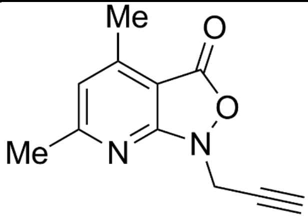
**17**	1-(4-fluorobenzoyl)-4,6-dimethylisoxazolo[3,4-*b*]pyridin-3(1*H*)-one	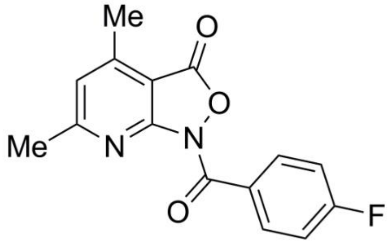
**18**	1-(3,4,5-trimethoxybenzyl)-4,6-dimethylisoxazolo[3,4-*b*]pyridin-3(1*H*)-one	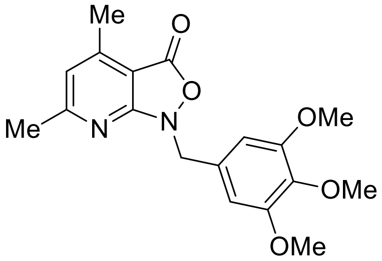
**19**	6-methoxy-1-methylisoxazolo[3,4-*b*]quinolin-3(1*H*)-one	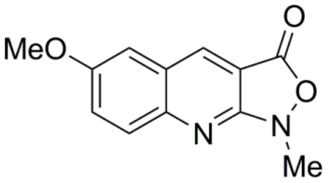
**20**	6-fluoro-1-methylisoxazolo[3,4-*b*]quinolin-3(1*H*)-one	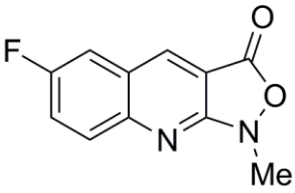
**21**	6-chloro-1-methylisoxazolo[3,4-*b*]quinolin-3(1*H*)-one	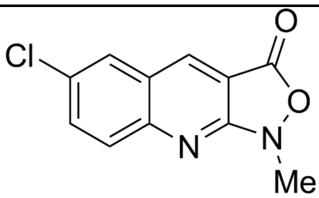
**22**	1,6,7-trimethylisoxazolo[3,4-*b*]quinolin-3(1*H*)-one	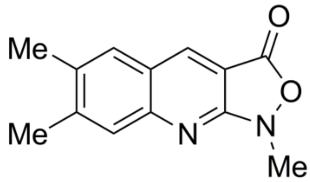

**23**	5,8-dimethoxyisoxazolo[3,4-*b*]quinolin-3(1*H*)-one	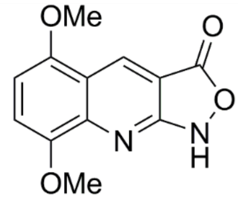
**24**	1,7-dimethylisoxazolo[3,4-*b*]quinolin-3(1*H*)-one	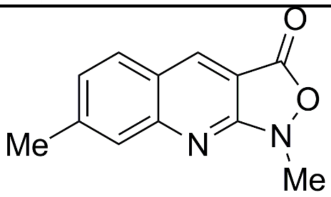
**25**	5,8-dimethoxy-1-methylisoxazolo[3,4-*b*]quinolin-3(1*H*)-one	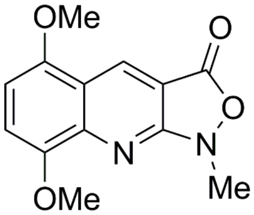
**26**	1,6-dimethylisoxazolo[3,4-*b*]quinolin-3(1*H*)-one	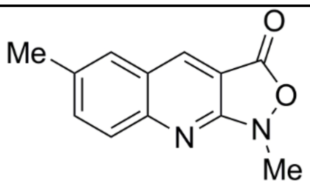

Table 2S. Minimum, maximum and mean value of log*P*-s calculated by different software

	mean log P	minimal log P	maximal log P
log P Chem Draw	1.88	-0.42	3.10
Clog P Chem Draw	2.44	0.11	4.10
vlogp	2.45	0.95	3.43
LogPC Spartan	2.65	0.69	4.47
LogPV Spartan	0.76	-0.71	4.72
miLogP	1.95	0.54	3.16
log KOWWIN	2.29	-0.04	3.94
AlogPs	2.26	0.72	3.67
AClogP	2.46	1.24	3.52
AlopgP	2.56	0.96	3.95
MlogGP	2.51	1.02	3.70
XlogP2	2.11	0.72	3.44
XlogP3	2.58	0.92	3.96

Table 3S. Retention data for the investigated pyrido- and quinolino-isoxazolones obtained from the Soczewiński–Wachtmeister's with statistical parameters

Stationary phase: C_8 bonded silica gel<br>Mobile phase: MeOH-Water								
No	R_M^0	σR_M^0	m	σm	R	R^2	F	s
1	1.496	0.102	-2.198	0.151	0.991	0.981	210.480	0.063
2	3.120	0.146	-3.730	0.217	0.993	0.987	296.732	0.091
3	2.162	0.092	-2.805	0.137	0.995	0.991	418.648	0.057
4	1.893	0.067	-2.593	0.100	0.997	0.994	672.229	0.042
5	2.929	0.096	-3.592	0.144	0.997	0.994	626.162	0.060
6	3.293	0.214	-4.063	0.318	0.988	0.976	163.499	0.133
7	1.791	0.121	-2.477	0.180	0.990	0.979	189.270	0.075
8	2.175	0.089	-2.772	0.132	0.995	0.991	438.375	0.055
9	2.834	0.156	-3.539	0.231	0.992	0.983	233.815	0.097
10	1.919	0.094	-2.558	0.140	0.994	0.988	334.492	0.059
11	3.477	0.106	-4.192	0.158	0.997	0.994	706.077	0.066
12	2.238	0.164	-2.094	0.244	0.973	0.949	73.798	0.101
13	2.988	0.065	-3.555	0.096	0.999	0.997	1357.867	0.040
14	0.991	0.058	-1.831	0.086	0.996	0.991	455.085	0.036
15	2.722	0.103	-3.391	0.153	0.996	0.992	489.391	0.064
16	1.951	0.075	-2.734	0.111	0.997	0.993	602.508	0.047
17	2.864	0.133	-3.579	0.198	0.994	0.988	326.728	0.083
18	2.808	0.097	-3.476	0.144	0.997	0.993	583.604	0.060
19	2.421	0.049	-3.023	0.072	0.999	0.998	1746.354	0.030
20	2.123	0.040	-2.780	0.059	0.999	0.998	2223.849	0.025
21	2.568	0.079	-3.182	0.118	0.997	0.995	732.630	0.049
22	2.907	0.058	-3.498	0.087	0.999	0.998	1632.503	0.036
23	2.123	0.027	-2.664	0.041	1.000	0.999	4242.300	0.017
24	2.431	0.031	-3.033	0.046	1.000	0.999	4333.181	0.019
25	2.557	0.036	-3.026	0.054	0.999	0.999	3161.924	0.023
26	2.461	0.089	-2.990	0.133	0.996	0.992	508.969	0.055

Table 3S. (continued)

Stationary phase: C_{18} bonded silica gel<br>Mobile phase: MeOH-Water								
No	R_M^0	σR_M^0	m	σm	R	R^2	F	s
1	2.771	0.163	-3.153	0.200	0.999	0.997	1183.611	0.019
2	3.702	0.213	-4.031	0.262	0.994	0.987	236.093	0.083
3	2.045	0.060	-2.414	0.074	0.999	0.997	1056.838	0.023
4	1.935	0.035	-2.440	0.043	1.000	0.999	3170.983	0.014
5	3.143	0.117	-3.561	0.144	0.998	0.995	609.047	0.046
6	3.012	0.142	-3.363	0.175	0.996	0.992	367.840	0.055
7	1.616	0.053	-2.087	0.066	0.999	0.997	1013.956	0.021
8	2.098	0.041	-2.465	0.050	0.999	0.999	2383.061	0.016
9	2.901	0.056	-3.345	0.069	0.999	0.999	2359.717	0.022
10	1.775	0.071	-2.186	0.088	0.998	0.995	620.512	0.028
11	3.041	0.132	-3.324	0.162	0.996	0.993	419.007	0.051
12	3.017	0.669	-3.633	0.823	0.931	0.867	19.491	0.260
13	3.175	0.084	-3.457	0.104	0.999	0.997	1108.349	0.033
14	0.945	0.263	-1.841	0.323	0.957	0.915	32.394	0.102
15	2.770	0.058	-3.111	0.071	0.999	0.998	1927.377	0.022
16	1.948	0.056	-2.641	0.068	0.999	0.998	1494.420	0.022
17	3.158	0.038	-3.649	0.047	1.000	0.999	5987.251	0.015
18	3.151	0.052	-3.743	0.064	1.000	0.999	3382.991	0.020
19	2.509	0.094	-2.888	0.116	0.998	0.995	618.312	0.037
20	2.314	0.104	-2.778	0.128	0.997	0.994	473.594	0.040
21	2.412	0.171	-2.718	0.210	0.991	0.982	167.234	0.066
22	3.028	0.131	-3.319	0.161	0.996	0.993	425.171	0.051
23	2.066	0.101	-2.569	0.125	0.996	0.993	425.453	0.039
24	2.731	0.131	-3.107	0.162	0.996	0.992	369.059	0.051
25	2.664	0.137	-2.980	0.169	0.995	0.990	310.187	0.054
26	2.771	0.163	-3.153	0.200	0.994	0.988	247.242	0.063

R the coefficient of correlation, R^2 the coefficient of determination, F the value of the Snedecor's F-test, s the standard estimation error, p value < 0.05.

Table 4S. Antifungal activity toward references *Candida* species [1–3]

No.	Candida albicans ATCC 10231	Candida glabrata ATCC 66032	Candida lusitaniae ATCC 34499	Candida parapsilosis ATCC 22019	Candida tropicalis ATCC 750
1	>128	>128	>128	>128	>128
2	>128	>128	>128	>128	>128
3	>128	>128	>128	>128	>128
4	>128	>128	>128	>128	>128
5	>128	>128	>128	>128	>128
6	100	>200	100	>200	>200
7	>200	>200	100	>200	>200
8	>200	>200	>200	>200	>200
9	50	50	50	50	50
10	>200	>200	>200	>200	>200
11	>200	>200	>200	100	>200
12	100	>200	100	>200	>200
13	*nt*	*nt*	*nt*	*nt*	*nt*
14	>200	>200	50	>200	100
15	100	>200	50	<6.2	25
16	100	>200	>200	<6.2	>200
17	50	100	25	<6.2	100
18	>200	>200	>200	100	>200
19	>128	>128	>128	>128	>128
20	>128	>128	>128	>128	>128
21	>128	>128	>128	>128	>128
22	>128	>128	>128	>128	>128
23	>128	>128	>128	>128	>128
24	>128	>128	>128	>128	>128
25	>128	>128	>128	>128	>128
26	>128	>128	>128	>128	>128

nt – not tested

1. Saczewski, J.; Fedorowicz, J.; Korcz, M.; Saczewski, F.; Wicher, B.; Gdaniec, M.; Konopacka, A. Experimental and theoretical studies on the tautomerism and reactivity of isoxazolo[3,4-b]quinolin-3(1H)-ones. *Tetrahedron* **2015**, *71*, 8975–8984.

2. Saczewski, J.; Fedorowicz, J.; Kedzia, A.; Ziolkowska-Klinkosz, M.; Jalinska, A. Synthesis and Antifungal Activity of Some 4,6-Dimethylisoxazolo[3,4- b]pyridin-3(1H)-one Derivatives. *Med. Chem. (Los. Angeles).* **2016**, *12*, 640–646.

3. Saczewski, J.; Kedzia, A.; Jalińska, A. New derivatives of 4,6-dimethylisoxazolo[3,4-b] pyridin-3(1H)-one: Synthesis, tautomerism, electronic structure and antibacterial activity. *Heterocycl. Commun.* **2014**, *20*, 215–223.

Table 5S. Cross-validation – analysis of variance summary for the TLC C_8 QSRR model.

Source of ariance	*SS*	*df*	*MS*	*F*	Prob. > *F*
Total	16.512	17	0.971	49.44	4.66E-6
Fit	16.121	8	2.015		
Residual	0.391	9	0.0408		

SS – sum of squares, *df* – degrees of freedom, *MS* – median square, *F* – Fisher test statistic.

Table 6S. Cross-validation – analysis of variance summary for the TLC C_{18} QSRR model.

Source of ariance	*SS*	*df*	*MS*	*F*	Prob. > *F*
Total	16.463	17	0.968	244.26	8.60E-9
Fit	16.354	8	2.044		
Residual	0.109	9	0.008		

SS – sum of squares, *df* – degrees of freedom, *MS* – median square, *F* – Fisher test statistic.

Table 7S. Cross-validation – analysis of variance summary for the HPLC $\log k_w$ QSRR model.

Source of ariance	*SS*	*df*	*MS*	*F*	Prob. > *F*
Total	16.429	17	0.966	31.56	2.62E-5
Fit	15.979	8	1.997		
Residual	0.451	9	0.063		

SS – sum of squares, *df* – degrees of freedom, *MS* – median square, *F* – Fisher test statistic.

Table 8S. Cross-validation – analysis of variance summary for the MEKC $\log k$ QSRR model.

Source of variance	*SS*	*df*	*MS*	*F*	Prob. > *F*
Total	16.731	17	0.984	19.54	4.47E-5
Fit	16.185	10	1.619		
Residual	0.546	7	0.083		

SS – sum of squares, *df* – degrees of freedom, *MS* – median square, *F* – Fisher test statistic.

Table 9S. List of molecular descriptors with coefficients values in PLS models built for RP-LC and MEKC.

TLC C_8 QSRR model				
No.	Name	Coefficients	Descriptor full name	Block
1	PJI2	0.09	2D Petitjean shape index	Topological indices
2	X5Av	0.12	average valence connectivity index of order 5	Connectivity indices
3	MATS7i	-0.03	Moran autocorrelation of lag 7 weighted by ionization potential	2D autocorrelations
4	GATS3p	0.08	Geary autocorrelation of lag 3 weighted by polarizability	2D autocorrelations
5	GATS5p	-0.21	Geary autocorrelation of lag 5 weighted by polarizability	2D autocorrelations
6	JGI2	-0.12	mean topological charge index of order 2	2D autocorrelations
7	SpMax2_Bh(s)	-0.11	largest eigenvalue n. 2 of Burden matrix weighted by I-state	Burden eigenvalues
8	SpMax3_Bh(s)	0.11	largest eigenvalue n. 3 of Burden matrix weighted by I-state	Burden eigenvalues
9	Mor19s	-0.01	signal 19 / weighted by I-state	3D-MoRSE descriptors
10	E2u	-0.24	2nd component accessibility directional WHIM index / unweighted	WHIM descriptors
11	E1i	0.24	1st component accessibility directional WHIM index / weighted by ionization potential	WHIM descriptors
12	HATS4u	-0.30	leverage-weighted autocorrelation of lag 4 / unweighted	GETAWAY descriptors
13	HATS4v	-0.26	leverage-weighted autocorrelation of lag 4 / weighted by van der Waals volume	GETAWAY descriptors
14	R3p	0.20	R autocorrelation of lag 3 / weighted by polarizability	GETAWAY descriptors
15	R4p	0.27	R autocorrelation of lag 4 / weighted by polarizability	GETAWAY descriptors
16	R7p+	0.42	R maximal autocorrelation of lag 7 / weighted by polarizability	GETAWAY descriptors
17	CATS3D_08_AL	0.15	CATS3D Acceptor-Lipophilic at lag 08	CATS 3D
TLC C_{18} QSRR model				
No	Name	Coefficients		
1	PJI2	0.04	2D Petitjean shape index	Topological indices
2	X5Av	0.27	average valence connectivity index of order 5	Connectivity indices
3	MATS2s	-0.06	Moran autocorrelation of lag 2 weighted by I-state	2D autocorrelations
4	SpMax3_Bh(s)	0.19	largest eigenvalue n. 3 of Burden matrix weighted by I-state	Burden eigenvalues
5	DISPp	-0.11	displacement value / weighted by polarizability	Geometrical descriptors
6	Mor10v	0.15	signal 10 / weighted by van der Waals volume	3D-MoRSE descriptors
7	Mor10p	0.11	signal 10 / weighted by polarizability	3D-MoRSE descriptors
8	E1e	0.08	1st component accessibility directional WHIM index / weighted by Sanderson electronegativity	WHIM descriptors
9	E2p	-0.20	2nd component accessibility directional WHIM index / weighted by polarizability	WHIM descriptors

Table 8S. (continued)

10	Kv	0.18	K global shape index / weighted by van der Waals volume	WHIM descriptors
11	HATS1e	-0.18	leverage-weighted autocorrelation of lag 1 / weighted by Sanderson electronegativity	GETAWAY descriptors
12	HATS4i	-0.10	leverage-weighted autocorrelation of lag 4 / weighted by ionization potential	GETAWAY descriptors
13	R4v+	-0.08	R maximal autocorrelation of lag 4 / weighted by van der Waals volume	GETAWAY descriptors
14	R3p	0.24	R autocorrelation of lag 3 / weighted by polarizability	GETAWAY descriptors
15	R4p	0.15	R autocorrelation of lag 4 / weighted by polarizability	GETAWAY descriptors
16	CATS3D_03_DL	0.01	CATS3D Donor-Lipophilic at lag 03	CATS 3D
17	CATS3D_08_AL	0.08	CATS3D Acceptor-Lipophilic at lag 08	CATS 3D
			HPLC	
No	Label	Coefficients		
1	ATSC3p	0.29	Centred Broto-Moreau autocorrelation of lag 3 weighted by polarizability	2D autocorrelations
2	MATS7i	-0.11	Moran autocorrelation of lag 7 weighted by ionization potential	2D autocorrelations
3	MATS2s	-0.03	Moran autocorrelation of lag 2 weighted by I-state	2D autocorrelations
4	SpMax2_Bh(s)	-0.19	largest eigenvalue n. 2 of Burden matrix weighted by I-state	Burden eigenvalues
5	SpMax3_Bh(s)	0.18	largest eigenvalue n. 3 of Burden matrix weighted by I-state	Burden eigenvalues
6	DISPi	-0.15	displacement value / weighted by ionization potential	Geometrical descriptors
7	E2u	-0.14	2nd component accessibility directional WHIM index / unweighted	WHIM descriptors
8	Ki	0.04	K global shape index / weighted by ionization potential	WHIM descriptors
9	HATS4u	-0.13	leverage-weighted autocorrelation of lag 4 / unweighted	GETAWAY descriptors
10	HATS4v	-0.05	leverage-weighted autocorrelation of lag 4 / weighted by van der Waals volume	GETAWAY descriptors
11	R4v+	-0.13	R maximal autocorrelation of lag 4 / weighted by van der Waals volume	GETAWAY descriptors
12	R3p	0.21	R autocorrelation of lag 3 / weighted by polarizability	GETAWAY descriptors
13	R4p	0.28	R autocorrelation of lag 4 / weighted by polarizability	GETAWAY descriptors
14	R7p+	0.18	R maximal autocorrelation of lag 7 / weighted by polarizability	GETAWAY descriptors
15	R4s	0.05	R autocorrelation of lag 4 / weighted by I-state	GETAWAY descriptors
16	R6s+	0.19	R maximal autocorrelation of lag 6 / weighted by I-state	GETAWAY descriptors
17	CATS3D_08_AL	0.20	CATS2D Acceptor-Lipophilic at lag 08	CATS 3D

Table 9S. (continued)

MECK				
No	Label	Coefficients		
1	VE1_Dz(p)	0.14	coefficient sum of the last eigenvector from Barysz matrix weighted by polarizability	2D matrix-based descriptors
2	Mor24s	-0.24	signal 24 / weighted by I-state	3D-MoRSE descriptors
3	E2u	0.52	2nd component accessibility directional WHIM index / unweighted	WHIM descriptors
4	E1e	0.24	1st component accessibility directional WHIM index / weighted by Sanderson electronegativity	WHIM descriptors
5	HATS1e	-0.56	leverage-weighted autocorrelation of lag 1 / weighted by Sanderson electronegativity	GETAWAY descriptors
6	HATS7p	0.06	leverage-weighted autocorrelation of lag 7 / weighted by polarizability	GETAWAY descriptors
7	R4v+	0.33	R maximal autocorrelation of lag 4 / weighted by van der Waals volume	GETAWAY descriptors
8	R4p	0.16	R autocorrelation of lag 4 / weighted by polarizability	GETAWAY descriptors
9	R4p+	0.17	R maximal autocorrelation of lag 4 / weighted by polarizability	GETAWAY descriptors
10	R6s+	0.31	R maximal autocorrelation of lag 6 / weighted by I-state	GETAWAY descriptors
11	CATS3D_08_AA	-0.49	CATS3D Acceptor-Acceptor at lag 08	CATS 3D
12	CATS3D_03_LL	-0.33	CATS3D Lipophilic-Lipophilic at lag 03	CATS 3D

Figure 1S. Optimization of the number of latent variables (LVs) for the consensus GA-PLS models for **A)** TLC with the C_8 plate, **B)** TLC with the C_{18} plate, **C)** HPLC logk_w, and **D)** MEKC log$k$ parameters. The optimal number of LVs is denoted in pink.

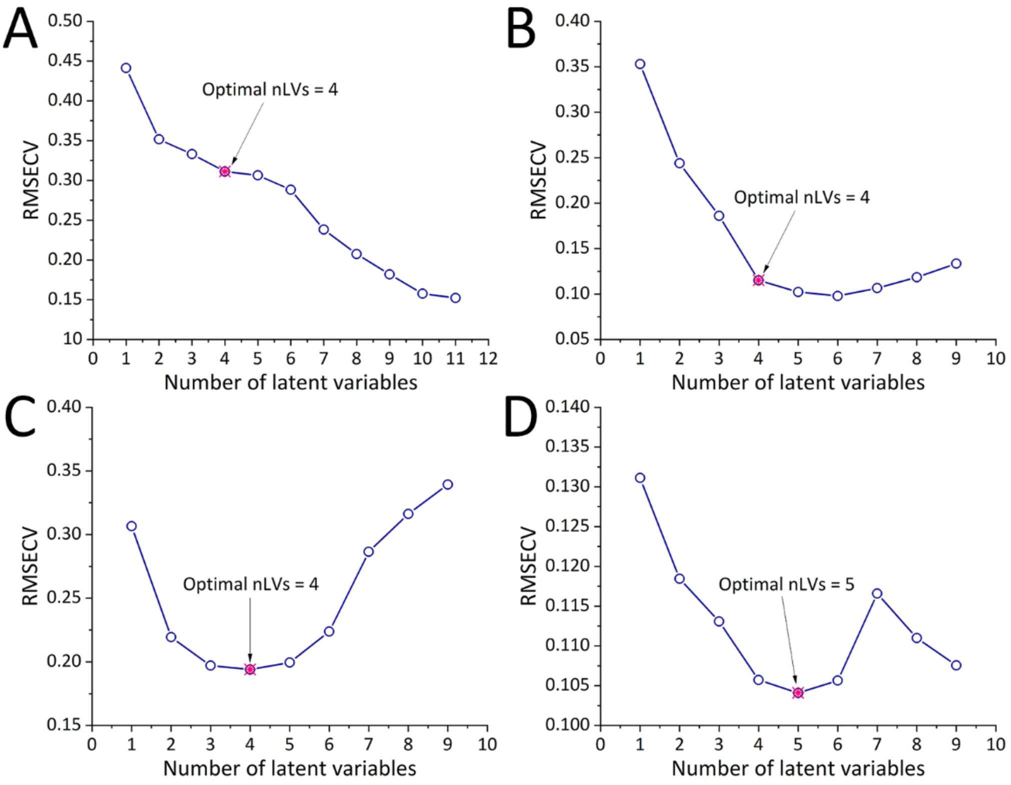

Figure 2S. Leave one out-cross validation (LOO-CV) predictive ability (on the training set) for **A)** TLC with the C_8 plate, **B)** TLC with the C_{18} plate, **C)** HPLC logk_w, and **D)** MEKC log$k$ parameters.

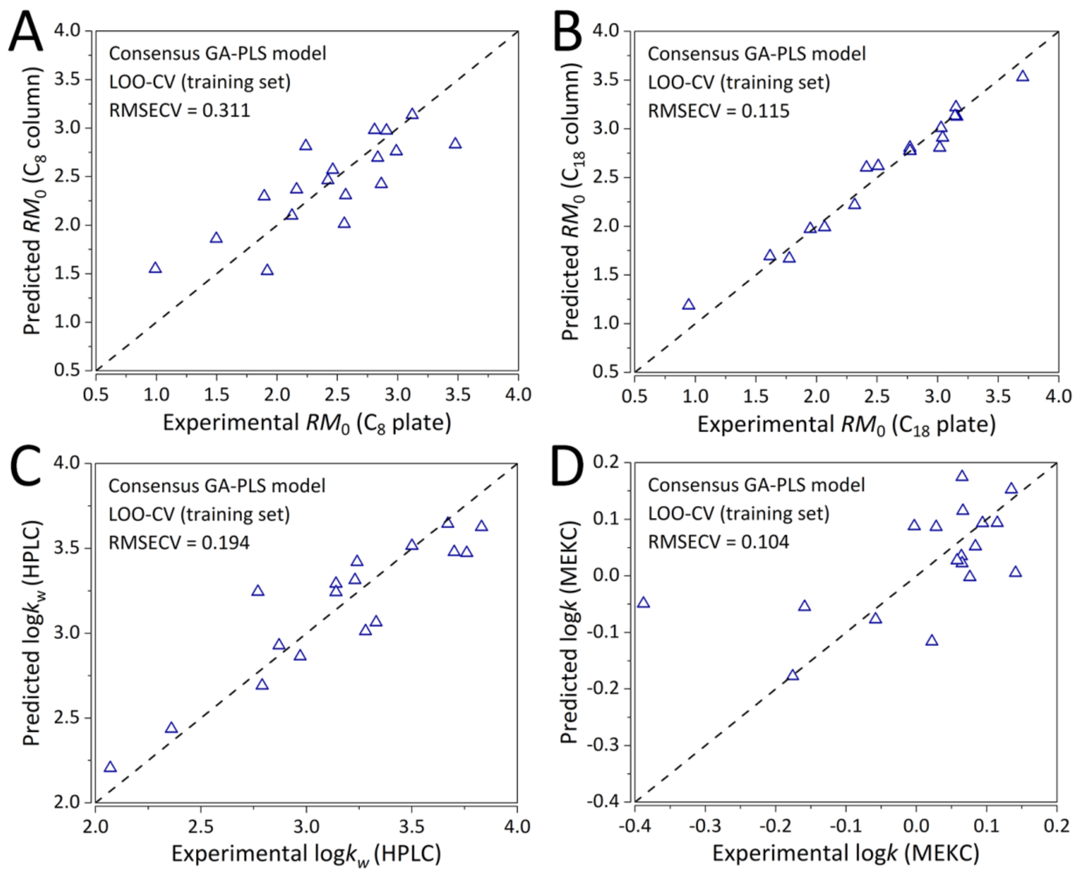

Supplement: Supplementary file 1 [file molecules-24-04311-s001.zip › molecules-644116-supplementary-final/SI_molecules_2019_14.11.2019.pdf]
